# Early steps of microglial activation are directly affected by neuroprotectant FK506 in both in vitro inflammation and in rat model of stroke

**DOI:** 10.1007/s00109-012-0925-9

**Published:** 2012-07-18

**Authors:** Malgorzata Zawadzka, Michal Dabrowski, Agata Gozdz, Barbara Szadujkis, Marcin Sliwa, Maciej Lipko, Bozena Kaminska

**Affiliations:** 1 Laboratory of Transcription Regulation, Department Cell Biology, Nencki Institute of Experimental Biology, 3 Pasteur Str., 02-093 Warsaw, Poland; 2 Laboratory of Transcription Regulation, Nencki Institute of Experimental Biology, 3 Pasteur Str., 02-093 Warsaw, Poland

**Keywords:** CNS inflammation, Microglia activation, Immunosuppressants, MAPK signaling, Gene expression profiling

## Abstract

Neuroprotective and/or neuroregenerative activity of FK506, its derivatives, and to a lesser extent cyclosporin A (CsA) in animal models of neurodegenerative diseases of different etiology have been reported. Here, we verified a hypothesis that the most likely mechanism of their neuroprotective action is inhibition of the early steps of inflammatory activation of microglia by interference with mitogen-activated protein kinase (MAPK) signaling. The effect of immunosuppressants on lipopolysaccharide (LPS)-induced changes in morphology, proliferation, and motility of rat primary microglial cultures was evaluated. FK506 and CsA directly inhibited LPS-induced microglia activation and inflammatory responses. While both drugs efficiently reduced the expression of iNOS and the release of nitric oxide, only FK506 strongly inhibited the expression of Cox-2 and secretion of the mature form of IL-1β. FK506 strongly reduced LPS-induced activation of MAPK, and its downstream signaling crucial for inflammatory responses. Comparative analysis of global gene expression in rat ischemic brains and in LPS-stimulated microglial cultures revealed many genes and signaling pathways regulated in the same way in both systems. FK506 treatment blocked a majority of genes induced by an ischemic insult in the cortex, in particular inflammatory/innate immunity and apoptosis-related genes. Microglia-mediated inflammation is considered as one of the most important components of brain injury after trauma or stroke; thus, effective and multifaceted blockade of microglial activation by FK506 has clinical relevance and potential therapeutic implications.

**Electronic supplementary material:**

The online version of this article (doi:10.1007/s00109-012-0925-9) contains supplementary material, which is available to authorized users.

## Introduction

Microgliosis is one of the most important components of poststroke neuroinflammation which is also accompanied by astrogliosis, and manifests in local inflammation, infiltration of immune cells, and activation of the adaptive immunity [1]. In response to brain injury or infection, intracellular signaling pathways are activated in microglia, which turn on inflammatory and antigen-presenting cell functions. Acute activation of microglia can contribute to neuronal damage by production of cytotoxic and inflammation mediators such as nitric oxide, reactive oxygen species, prostaglandin products, pro-inflammatory cytokines IL-1β, tumor necrosis alpha (TNF-α), IL-2, IL-6, or by autoimmune mechanisms [2, 3]. Clinical and experimental data showed a detrimental role of inflammatory cytokines in injured brain, and inhibition of IL-1β and TNF-α activity with neutralizing antibodies or soluble receptors decreased neuronal damage in animal models of stroke [4, 5].

Numerous studies have reported neuroprotective and/or neuroregenerative activity of immunosuppressant FK506, its derivatives, and to a lesser extent cyclosporin A (CsA) in animal models of neurological diseases of different etiology: traumatic brain injury, spinal cord injury, optic nerve crush, antiretroviral toxic neuropathy, rodent models of Parkinson's disease, and stroke [6–8]. Therapeutic time window of FK506 is narrow: 2–3 h in the rat middle cerebral artery occlusion (MCAo) model. In the nonhuman primate stroke model, drug administration 2 h after the ischemic insult significantly reduced neurological deficits and infarct volumes in the cerebral cortex. Administration of FK506 4 h after the insult showed significant amelioration of neurological deficits, suggesting a therapeutic time window for FK506 comparable with 3–4.5 h reported for a tissue plasminogen activator (rt-PA, alteplase) [9]. FK506 exerts multiple types of action, such as inhibition of apoptotic and necrotic cell death in cultured neurons, attenuation of leukocyte accumulation, and attenuation of glia activation [10, 11]. It suggests the drug's interference with cellular processes which are common to many neuropathological conditions, and a mechanistic pathway for its neuroprotective effect in vivo needs to be explored.

We have previously demonstrated that FK506 administered 1 h after transient MCAo reduced microglia activation as well as hypertrophy of astrocytes in the ischemic penumbra, ameliorating brain damage and neurological deficits [12]. Recently, we performed global gene expression profiling and the functional analysis of the genes affected after MCAo in rats revealed that the ten significantly overrepresented gene categories include “response to wounding” and “inflammatory response” [13]. This may represent gene induction in activated microglia as the early expression of inflammatory cytokines in the ischemic brain has been demonstrated predominantly in microglia [12]. Thus, we hypothesized that FK506 may act by blocking initiation of microglial inflammatory responses.

In the present study, we demonstrate that FK506 is a potent inhibitor of microglial activation and production of inflammation mediators. It likely interferes with activation of mitogen-activated protein kinases (MAPK) and nuclear factor kappa B (NFκB) signaling pathways, crucial for regulation of inflammatory gene expression. We identified an inflammatory gene signature common for lipopolysaccharide (LPS)-stimulated microglial cultures and ischemic brain, and demonstrated the inhibitory effect of FK506 on the expression of numerous genes related to inflammatory response and cell death in a rat model of stroke. We postulate that pharmacological intervention with FK506 may be a good therapeutic strategy targeting the inflammatory component of stroke.

## Methods

### Cell culture and treatment

Primary cultures of rat microglia were prepared as described [14]. Briefly, cells were isolated from cerebral cortices and plated at the density of 3 × 10^5^ cells/cm^2^ in culture medium (DMEM/Glutamax/high glucose, Gibco, 10 % FBS, 100 U/mL penicillin, and 0.1 mg/mL streptomycin) on poly-l-lysine-coated culture flasks. After 2 weeks of culture, the loosely adherent microglial cells were recovered from confluent glial cultures by a mild shaking and centrifugation. Cells were suspended in the culture medium and plated at density of 3 × 10^5^ cells/cm^2^ onto 24-well plates or 60-mm dishes. Nonadherent cells were removed after 30 min by changing the medium. Adherent cells (>96 % positive for isolectin B_4_) were incubated for 48 h. Cells were stimulated with 100 ng/mL LPS from *Salmonella enteritidis* (Sigma, Germany). Compounds were added 30 min before LPS stimulation. FK506 (LC Laboratories, Woburn, MA, USA) was diluted in ethanol; control cells received an equal amount of ethanol. Cyclosporin A (Sandimmune, Novartis, Basel, Switzerland) was diluted in a culture medium.

### Immunocytochemistry

For immunocytochemistry, cells were fixed with 4 % paraformaldehyde at indicated time points after treatment. Next, cells were incubated with fluorescein isothiocyanate (FITC)-conjugated isolectin B_4_ from *Bandeiraea simplicifolia* (10 μg/mL, Sigma, Germany) for 4 h at room temperature and counterstained with DAPI (4′,6-diamidino-2-phenylindole, Sigma, Germany, 10 μg/mL). The effects of the compounds were monitored by fluorescent microscopy (450–490 nm). Ten randomly distributed visual fields were captured for each experimental condition used in three independent experiments. Cell body area was measured using ImageJ software (NIH, http://rsb.info.nih.gov/ij/).

### Proliferation and cell motility assays

Microglial proliferation was determined by BrdU incorporation assay in cell cultures exposed to various treatments (untreated, 100 ng/mL LPS alone, or with immunosuppressant). After 18 h, BrdU (10 μM) was added to the culture medium for 6 h, and subsequently, cells were fixed, and the level of BrdU incorporation was estimated according to the manufacturer’s protocol (Roche, Mannheim, Germany).

For motility assay, cells were plated on a 35-mm Petri dish, and the cultures were gently scratched using a 100-μl pipette tip. After washing with phosphate-buffered saline (PBS), cells were left untreated or were exposed for 6 h to LPS alone or in combination with 5 μM FK506 or CsA. The cells were fixed and cell nuclei were stained with DAPI. Cells from more than three representative fields for every treatment were counted using ImageJ software.

### Evaluation of IL-1β and nitric oxide production

Microglia were stimulated with LPS alone or in combination with immunosuppressants. Twenty-four hours after stimulation, the culture media were collected, centrifuged at 500×*g* for 10 min, and the supernatants were used for determination of NO and cytokine production. NO production was assessed with the NO release assay (Active Motif, Rixensart, Belgium). IL-1β production was measured by ELISA using rat-specific antibody pairs and rat protein standards in accordance with the manufacturer's instructions (R&D Systems, Wiesbaden, Germany). Cytokine levels were calculated as nanograms per milliliter and expressed as a percentage of the IL-1β release in control cultures.

### Protein isolation, electrophoresis, and detection

Whole-cell protein extracts were separated on SDS–PAGE and transferred onto a nitrocellulose membrane (Amersham Biosciences, Germany). Antibodies recognizing phosphorylated forms of p38, p44/42 MAP kinase (ERK1/2), JNK, MAPKAP-2, c-Jun, and IκB as well as Cox-2 (1:1,000) were purchased from Cell Signaling Technology (Beverly, MA, USA), inducible NO synthase (iNOS; diluted 1:2,000) was from BD Biosciences (Bedford, MA, USA) and IL-1β (diluted 1:1,000) from Santa Cruz Biotechnology (Santa Cruz, CA, USA). Immunocomplexes were visualized by using ECL (Amersham). To verify equal amounts of protein loading, the membranes were reprobed with anti-β-Actin antibody (diluted 1:2,000, from Oncogene Research Products, MA, USA).

### Transient middle cerebral artery occlusion and RNA extraction

MCAo was induced for 90 min with the intraluminal filament method as described [12, 13]. The procedure resulted in ischemic damage in the frontoparietal somatosensory cortex, striatum, and other areas such as hypothalamus, corresponding to the territory supported by the middle cerebral artery (MCA). FK506 (Tacrolimus, Fujisawa, UK) was administrated intravenously at 1 mg/kg body weight 60 min after MCAo. FK506 significantly reduced tissue damage in the cerebral cortex by 63 % as assessed by TTC staining [12]. Rats were lethally anesthetized at 12 h of reperfusion, brains were rapidly removed, and the same dorsolateral parts of the cerebral cortex containing MCA territory were dissected. Total RNA was extracted using TRI reagent (Sigma, Germany) and cleaned using RNeasy Total RNA kit (Qiagen, Germany). The amount and quality of the RNA were determined by capillary electrophoresis with the Bioanalyzer 2100 (Agilent Technologies).

### Microarray gene expression profiling

Comparative analysis of global gene expression was performed on two experimental paradigms: LPS-stimulated primary microglial cultures (six independent cultures for the control conditions and four RNA samples for LPS-treated cultures) and brain ischemia induced by middle cerebral artery occlusion (sham-operated (S) and MCAo-subjected rats with (F) or without (M) treatment with FK506, *n* = 3).

Biotin-labeled cRNAs were synthesized with the Affymetrix IVT labeling kit. Fragmented cRNA was hybridized first to a control microarray and then, after sample quality evaluation, was hybridized to the Affymetrix GeneChip Rat Genome 230-2.0 Gene Chips (31,042 probe sets including 28,000 rat genes).

Microarray data were preprocessed with the MAS 5.0 algorithm, as implemented in the "affy" R Bioconductor package [15]. Only consistently detected probesets in all hybridizations for at least one condition in a given experiment (call: Present or Marginal) were mapped to Ensembl 57 gene identifiers (gene_stable_id). For each gene, we computed a single average intensity profile from the profiles of all the probesets mapped to it. The resulting average profile was then log_2_-transformed and used in statistical analysis and visualization.

In the MCAo model, we used one-way ANOVA to identify the genes that changed expression among the three conditions (S, M, and F). We used unpaired Student’s *t* test, with Welch’s approximation for the degrees of freedom, to identify genes with significantly different mean expression between the contrasted groups (S vs. M, M vs. F). In the in vitro experiment, we used paired *t* test to identify genes with mean difference in expression, between the control and LPS for matched microglia preparations, significantly different from zero. For the false discovery rate (FDR) analysis, the lists of *p* values were imported into R statistical environment (http://www.R-project.org), and the *q* values [16] were calculated using the R “qvalue” package. To identify functional Gene Ontology categories associated with the observed changes in expression, the lists of gene symbols (Ensembl display_id) of the genes with the respective *t* test *p* value < 0.05 were ranked on the difference in log_2_ expression, either M minus S, or F minus M, and for LPS microglia minus control, and analyzed using Rank GOstat [17] with the default options (Wilcoxon signed rank test, Benjamini false discovery rate correction for multiple testing).

The datasets from both experiments were submitted to ArrayExpress, and are available with the following accession numbers: E-MEXP-2222 (MCAo) and E-MEXP-2466 (microglia).

### Statistical analysis

In vitro experiments were performed on at least three independently derived primary microglial cultures, in triplicates. Statistical analysis was carried out using one-way analysis of variance (ANOVA) followed by post hoc Newman–Keuls test with STATISTICA software (StatSoft, Tulsa, OK, USA). Statistical significance was determined at three levels: *p < 0.05, **p < 0.01, and ***p < 0.001. The results are expressed as the mean ± standard deviation (SD) or the mean ± standard errors of the mean (SEM) is indicated.

## Results

### Immunosuppressants inhibit morphological changes and motility of activated microglia

**Fig. 1:**
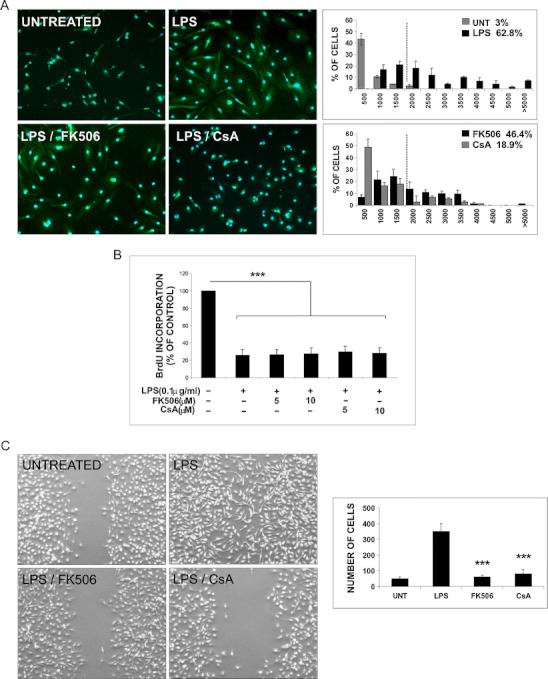
FK506 and CsA attenuate LPS-induced transformation of microglia. **a** Primary microglial cultures were prepared as described and kept for 48 h after plating to silence microglia. Morphological alterations of microglia untreated or exposed to 100 ng/mL LPS alone or with immunosuppressants (at concentration of 5 μM) for 24 h were visualized by immunofluorescence. Cells were stained with FITC-conjugated isolectin B_4_ (green) to visualize cell morphology followed by staining with DAPI for nuclei visualization (*blue*); ×20 objective. Stained cells with a specified range of size and optical density above fixed threshold value were automatically recognized and counted. Cell body area of microglial cells was measured, and the percentage of cells with body area >2,000 μm^2^ (activated cells) was calculated. *Left panel* shows the histograms representing the distribution of microglial cells body area (square micrometers) for all analyzed experimental conditions. Data are expressed as means ± SD from ten fields for all treatments in three independent experiments. **b** FK506 and CsA do not inhibit LPS-induced growth arrest of microglial cells. Microglial proliferation was determined with BrdU incorporation assay 24 h after treatment. The results are means ± SEM of three independent experiments, each in triplicate. **c** Immunosuppressants affect migration of activated microglia. Confluent microglial cultures were scratched with a plastic 100-μL tip, washed with PBS, and incubated for 5 h under given conditions. Cells migrating to a cell-free area without the treatment, after addition of 100 ng/mL LPS alone or with immunosuppressants (5 μM), were visualized by phase-contrast microscopy (magnification ×4). *Left panel* shows quantitative evaluation of motility/migration of microglia treated with LPS alone or with immunosupressants at 6 h time point. Cells from five fields between scratch edges along the line were counted. Results represent means ± SD from two independent experiments each in triplicate

In response to LPS, microglia undergo activation, which includes morphological transformation, increased motility, phagocytosis, and inflammatory gene expression. We analyzed an influence of FK506 and CsA on LPS-induced changes in morphology and behavior of the rat primary microglial cultures. Untreated microglial cells have bipolar or rod-shape morphology with few short processes (3 % of cells with 2,000 μm^2^ or larger cell body area), and exposure to LPS for 24 h resulted in cell body enlargement (62 % of cells with ≥2,000 μm^2^ cell body area) and amoeboid morphology. Treatment with immunosuppressants reduced LPS-induced morphological transformation of microglia by 26 % (in case of 5 μM FK506) or 70 % (5 μM CsA) (Fig. 1a). The inhibitory effect was more prominent at higher drug concentrations (10 μM FK506 and CsA, data not shown).

LPS inhibited microglial cell division in both mixed astrocyte/microglia cultures and in microglial cultures. To study if immunosuppressants could affect LPS-induced growth arrest, we assessed microglial proliferation (by measuring of BrdU incorporation) 24 h after treatment with LPS alone or with drugs. FK506 and CsA did not interfere with LPS-induced growth arrest, as shown in Fig. 1b.

Motility of microglial cells was examined with a scratch assay. The untreated microglial cells exhibit weak motility, only few cells migrated into the scratch area. Prominent induction of cell migration was observed in cultures treated with LPS. Immunosuppressants at concentration of 5 μM completely abolished LPS-induced cell motility (Fig. 1c).

### FK506 and CsA differentially modulate LPS-induced production of inflammation mediators

**Fig. 2:**
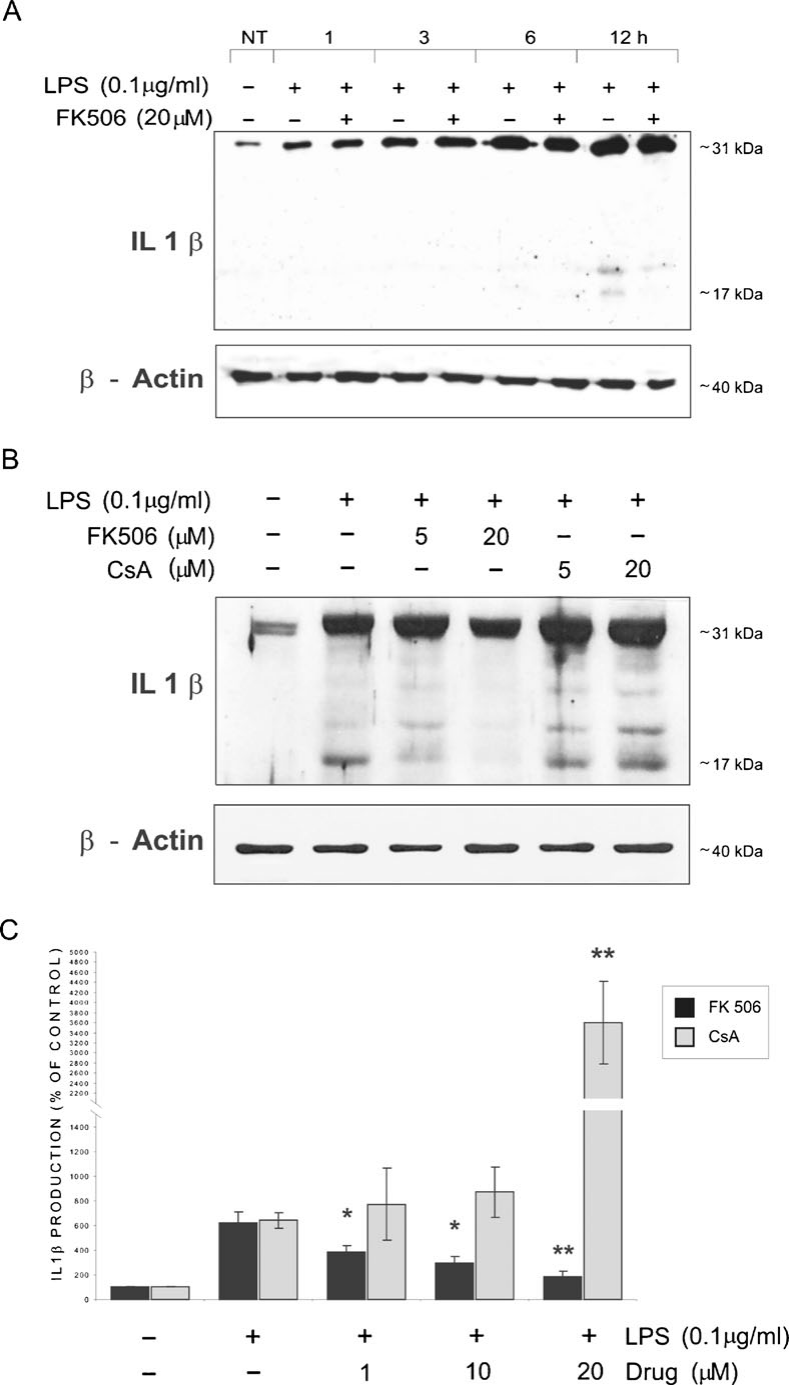
FK506 reduces LPS-induced production of IL-1β by microglial cells. **a** Kinetics of LPS-induced production of IL-1β. Immunoblot shows the levels of pro-IL-1β (31 kDa) and mature IL-1β (17 kDa) protein in extracts from microglial cells at different times after treatment with 100 ng/mL LPS. β-Actin detection ensured an equal protein loading. Similar results were observed on three independently derived microglial cultures. **b** Attenuation of LPS-induced production of the mature form of IL-1β in cells exposed to 5 and 20 μM FK506 for 12 h. **c** Effects of immunosuppressants on IL-1β secretion by LPS-stimulated microglial cultures evaluated by ELISA assay at 12 h after treatment. Results are expressed as percentage of the IL-1β release obtained in untreated cells; means ± SEM from three experiments (each in triplicate) are presented

We evaluated effects of immunosuppressants on the levels of IL-1β, NO, and Cox-2 produced by microglia. IL-1β protein level was assessed by Western blotting and ELISA. Increase of pro-IL-1β (31 kDa) level was detected in microglial cells 1 h after LPS addition and gradually elevated up to 12 h posttreatment (Fig. 2a), when the mature form of IL-1β (17 kDa) was detected. Pretreatment with FK506 reduced accumulation of the mature IL-1β in a dose-dependent manner (Fig. 2b). The results were corroborated by ELISA. Untreated cells produced 0.24 ± 0.03 ng/mL IL-1β, while upon activation the cytokine concentration reached 1.5 ± 0.2 ng/mL (Fig. 2c). Treatment with 20 μM FK506 decreased LPS-dependent cytokine secretion to 0.44 ± 0.1 ng/mL. In contrast, low doses of CsA had no effect on IL-1β secretion, while 20 μM CsA strongly elevated IL-1β secretion up to 8.71 ± 1.98 ng/mL.

**Fig.3:**
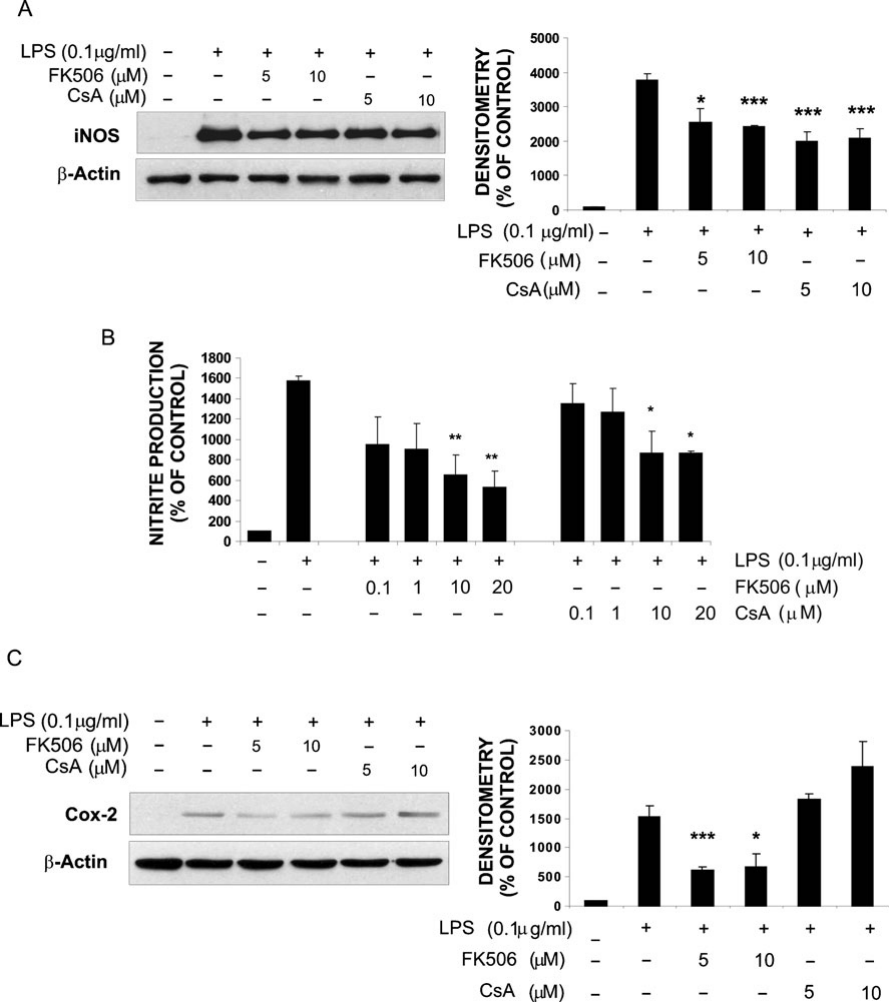
Immunosuppressants affect LPS-induced production of inflammatory mediators in rat microglial cultures. **a** Immunoblot shows that FK506 and CsA reduce the level of iNOS protein in microglial cells exposed for 24 h to 100 ng/mL LPS alone or together with FK506 or CsA (5 or 10 μM). Densitometry of immunoblots from three independent experiments was performed. The level of iNOS expression was normalized to β-actin content. All values are related to untreated controls and presented as the mean values ± SEM. **b** Nitric oxide production was determined as nitrite concentration in cell culture supernatants and expressed as percentage of the release by untreated cells. Results represent means ± SEM obtained from three independently derived cultures (each in triplicate). **c** FK506 (but not CsA) reduces LPS-induced expression of Cox-2 protein in microglial cultures. Densitometry of immunoblots from three independent experiments was performed. The level of Cox-2 expression was normalized to β-actin content, related to controls and presented as means ± SEM

The iNOS expression was significantly upregulated in LPS-activated microglia (Fig. 3a), and accordingly, NO production increased from 1.43 ± 0.57 μM in untreated cells up to 22.6 ± 0.82 μM in LPS-stimulated cultures (15.8-fold induction). Treatment with CsA or FK506 significantly decreased LPS-induced iNOS expression and NO production (Fig. 3a, b). FK506 was more potent than CsA and reduced the LPS-induced NO production by 58.42 and 66.34 % at the concentration of 10 and 20 μM, respectively, while CsA treatment resulted in 44.89 and 45.22 % reduction.

Cox-2 expression strongly upregulated by LPS treatment (Fig. 3c) was reduced by 5 and 10 μM FK506. In contrast, 5 μM CsA had no effect and at the higher dose increased the Cox-2 protein expression. Densitometry of immunoblots from three independent experiments confirmed reduction of LPS-induced Cox-2 protein expression in FK506- but not CsA-treated cultures.

### FK506 affects MAPK signaling in microglia

**Fig.4:**
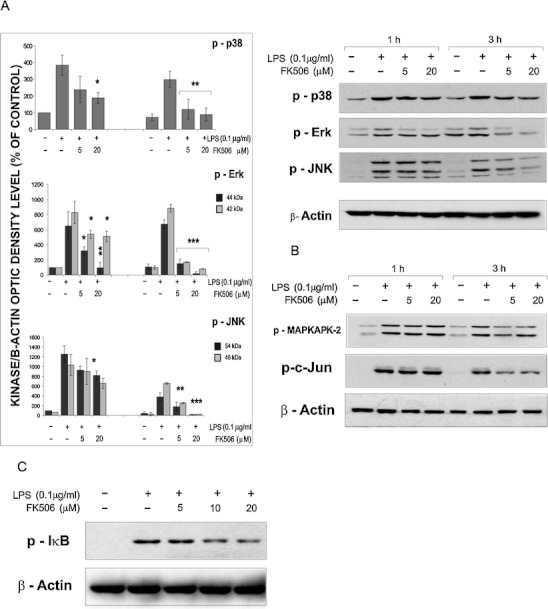
FK506 interferes with signal transduction triggered by LPS in microglial cultures. Immunoblots show the levels of phosphorylated forms of MAP kinases (**a**) their substrates, MAPKAPK2 and c-Jun (**b**) and IκB (**c**) Western blot analyses were performed with total extracts from microglial cultures treated with LPS alone or with FK506. Similar results were obtained in four independent experiments. The level of phosphorylated MAPK was quantified by densitometry, related to the β-actin level and then to values obtained for untreated microglia. Results are presented as means ± SEM from three independent experiments

Three MAPKs were activated shortly after stimulation of microglial cultures with LPS, as shown by Western blotting (Fig. 4a). Treatment with 5 and 10 μM FK506 decreased the levels of LPS-induced phosphorylation of MAPK at 1 and 3 h. Densitometry of immunoblots from four independent experiments demonstrated reduction of MAPK phosphorylation in FK506-treated microglial cultures. Concomitantly, LPS-triggered phosphorylation of downstream substrates of p38 and JNK, respectively MAPKAPK-2 and c-Jun, was also decreased by immunosuppressants (Fig. 4b). Moreover, pretreatment with FK506 or CsA inhibited LPS-induced activation of NFκB, a key transcriptional regulator of inflammatory response, as assessed by detection of the phosphorylated form of IκB (Fig. 4c). Our results indicate that FK506 impairs activation of MAPK and NFκB signaling in microglial cells that may result in blockade of inflammatory responses.

### Inflammation-related genes induced in ischemic brain and LPS-stimulated microglia are blocked by FK506

Since we demonstrated that the expression of crucial inflammatory factors such as IL-1β, IL-6, and TNF-α reached its maximum in the ipsilateral cortex at 12 h of reperfusion [12], global gene expression profiling in ischemic cortex has been performed 12 h after MCAo, MCAo followed by FK506 treatment, or in sham-operated animals, microglial cells were harvested 6 h of LPS stimulation (100 ng/mL), at the time point when the expression of inflammation-related genes was upregulated in vitro [18]. For comparisons of the sets of genes regulated by the studied treatments, we choose a uniform *p* value threshold of 0.05 in order to achieve gene sets of comparable sizes for each treatment. In the ischemic cortex, 1,803 genes significantly changed expression, with the cutoff at *p* value 0.05, FDR *q* value 0.15, among the 10,404 reliably detected genes. In LPS-treated microglia, 4,949 genes significantly changed its expression, with the cutoff at *p* value 0.05, FDR *q* value 0.03, among the 9,147 reliably detected genes.

**Fig.5:**
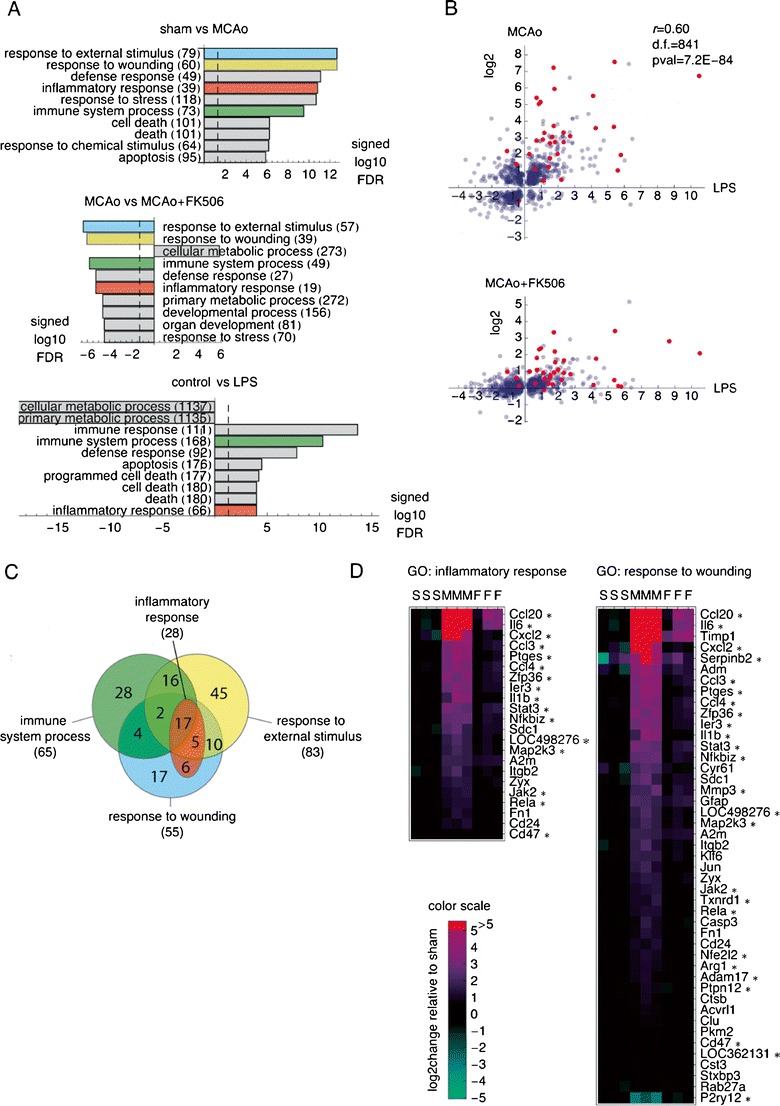
FK506 blocks upregulation of numerous inflammation-related genes in the ischemic cortex. **a** Gene Ontology “biological process” terms most significantly associated with the changes in gene expression induced by MCAo in the ischemic cortex, with or without FK506 treatment, and by LPS activation of microglia in vitro. Analysis was performed on the remote RankGOStat server, with ranked lists of log_2_ changes in expression of the genes with the respective *t* test *p* value < 0.05 used as the input files, and RGD selected as the target database. For the plot, the FDR-corrected association *p* values were log10-transformed and signed, i.e., multiplied by +1 or −1 to reflect association with increased or decreased expression, respectively. Numbers of genes in each GO category are given in *parentheses*. **b** Comparison of log_2_ changes in gene expression induced in microglia by LPS and after MCAo for the genes significantly regulated in both systems (*top panel*). The changes of the same genes as in the *top panel* are compared to the changes induced by MCAo in the animals that received FK506 (*bottom panel*). *Red dots* represent gene assigned to GO: inflammatory response. **c**. Venn diagram of the genes affected by FK506 treatment assigned to GO categories: “response to external stimulus,” “response to wounding,” “immune system process,” and “inflammatory response.” A newer local version of the GO database was used than by the RankGOStat server, resulting in larger numbers of genes assigned to the same GO terms than in (**a**). For details of the gene names and the expression data, see ESM Table 1. **d** Heat maps of expression profiles of the genes in GO catagories: “response to wounding” and “inflammatory response” that were regulated by FK506. The genes were sorted on the change in expression induced by MCAo (as compared to sham). The genes that were also significantly regulated during microglia activation with LPS are marked with an *asterisk*

Strong and consistent changes in the expression of similar sets of genes were observed in both systems. Analysis of the ranked list of genes that significantly changed expression (*p* < 0.05) after MCAo, and—separately—in LPS-treated microglia, revealed that in both experimental systems, the most affected genes represent similar Gene Ontology (GO) categories, including: “immune system process” and “inflammatory response” (Fig. 5a). Focusing only on genes that significantly changed expression in both models revealed 843 such genes (with *t* test *p* values of <0.05 in both datasets).

The top panel of Fig. 5b shows relative changes in gene expression induced by LPS in microglial cultures, and by stroke in the ischemic cortex, plotted against each other on the log_2_ scale. The log_2_ changes in gene expression in both systems were positively correlated (*r* = 0.60, *df* = 841, *p* value = 7.2, 10–84). The counts of the genes in each quadrant of the top panel of Fig. 5b (a contingency table) are given in Electronic supplementary materials (ESM) Table 3. Given these data, we asked if induction of a gene by LPS in the microglia in vitro predicts induction of the same gene in the cortex following the MCAo. As it turned out, there is a highly significant association between induction in both systems (Fisher’s exact test *p* value = 1.3, 10–47). When the induction by LPS was used to predict induction following the MCAo, for all the genes regulated in both systems, the sensitivity and specificity of prediction were 68 and 85 %, respectively. However, when the analysis was performed only for the 33 genes that were regulated in both systems and additionally assigned to the GO: inflammatory response term, the sensitivity and specificity of the prediction rose to 94 and 100 %, respectively. Thus, for the inflammatory response genes, upregulation by LPS in microglia in vitro accurately predicts upregulation following MCAo.

Majority of those genes, namely 615, were regulated in the same direction in both systems (dots in the I and III quadrant). Remarkably, 392 genes were upregulated in both systems (dots in the I-st quadrant). Thus, we identified a set of 392 genes upregulated in microglial cultures by LPS treatment that were also upregulated following MCAo. For those genes, the fold changes of expression were similar in both systems (dots scattered around the diagonal *Y* = *X*), and many of them were assigned to the GO category “inflammatory response” (red dots in Fig. 5b). It is likely that those genes were expressed in the same cell type, namely microglia.

FK506 treatment greatly reduced the changes in gene expression induced by MCAo, in particular for the genes that were most strongly induced by MCAo (Fig. 5b, compare top and bottom panel). In the FK506-treated animals, 1,173 genes had significantly different expression, at *p* < 0.05, FDR *q* value = 0.28, when compared to their expression in the control group (MCAo). Interestingly, most genes induced in ischemic cortex (as compared to sham-operated animals) were downregulated by FK506 treatment. The functional GO categories represented among the genes most strongly downregulated by FK506 were the same as the categories induced by MCAo and included: “response to external stimulus,” “response to wounding,” “immune system process,” and “inflammatory response” (Fig. 5a). Many of the genes affected by the FK506 treatment are assigned to several of these categories (Fig. 5c). Two categories, namely the “inflammatory response” and “immune system process,” were also induced by the LPS treatment (Fig. 5a). Validation of microarray data by qPCR in independent samples of differently stimulated microglia (*n* = 3) and in vivo experiments (*n* = 3–5 rats) is provided in ESM Fig. 2. The expression of nine genes, representing the most important targets for FK506 action, chosen on the basis of microarray analysis, has been verified. We confirmed the significant inhibition of inflammation-related gene expression (*ccl20*, *il6*, *cxcl2*, *ccl3*, *ccl4*, *il1*β, and *stat3*) by FK506 treatment (ESM Fig. 2A). Moreover, we validated the same effect of FK506 on LPS-stimulated microglia (ESM Fig. 2B). In both experimental paradigms, FK506 did not significantly affect *bdnf* expression, as we have previously reported [12]. The results achieved are in accordance with our previously demonstrated data that FK506 administered 1 h after transient MCAo ameliorated brain damage, reduced microglia activation as well as production of pro-inflammatory cytokine IL-1β in the ischemic penumbra (representative images of FK506 neuroprotective and anti-inflammatory effect are provided in ESM Fig. 3).

## Discussion

The main finding of this study is that FK506 restrains acute inflammatory responses in cultured microglial cells as well as in the ischemic brain. FK506 reduced LPS-induced activation of MAPK and NFκB signaling pathways, affecting crucial steps of microglia activation. This results in inhibition of morphological transformation, motility, and expression of inflammation mediators: IL-1β, Cox-2, and iNOS in inflammatory microglia. CsA applied at similar doses mimicked most of FK506 effects but failed to inhibit LPS-induced IL-1β production and Cox-2 expression. We observed upregulation of IL-1β secretion in cultures treated with 20 μM CsA, likely reflecting release of cellular content during CsA-induced cell death as microglial viability was reduced by concomitant treatment with LPS and 20 μM CsA (MTT metabolism test, data not shown). Also in naïve microglia, the mature IL-1β production was upregulated by 20 μM CsA (ESM Fig. 1). Cox-2 and iNOS expression was barely detectable under basal conditions, and the treatment of naïve, microglial cells with immunosuppressants did not affect their expression.

Our findings emphasize that FK506 is an efficient modulator of inflammatory responses in activated microglia in vitro and likely in vivo. Gene expression profiling revealed that FK506 attenuates global upregulation of “injury” and “inflammation-related” genes in the ischemic brain. Most significantly affected GO functional categories: “response to external stimulus,” “response to wounding,” and “inflammatory response” could reflect contribution of microglial expression to global genomic responses. The expression of a majority of upregulated inflammation-related genes was almost completely inhibited in FK506-treated ischemic animals.

Interestingly, FK506 strongly affected production of the mature IL-1β and cytokine secretion in microglial cultures. In response to LPS, newly synthesized IL-1β remains mostly cell associated and requires proteolytical processing by a cysteine protease IL-β-converting enzyme (caspase-1) to generate an active cytokine [19]. Inhibition of externalization or/and proteolytic processing of pro-IL-1β in FK506-treated microglial cultures could be a novel mechanism of drug action. However, this may not be a primary event, but a consequence of halting microglia activation. Inhibition of IL-1β activity or signaling with antibodies neutralizing cytokines or soluble cytokine receptors decreased neuronal damage in animal models of stroke [20]. Promising results of IL-1 receptor antagonist delivered peripherally were observed in stroke patients [5].

We show that LPS-induced upregulation of iNOS expression and NO production were inhibited strongly by FK506 and less potently by CsA. Previous results on the macrophage cell line RAW264.7 [21] or murine J774 macrophages suggested that 1–5 μg/mL CsA reduced iNOS expression and NO production, while FK506 was not [22] or less effective [23]. Both immunosuppressants inhibited LPS-induced NO production in rat peritoneal macrophages [24] that is in agreement with our results. We demonstrate that FK506 (but not CsA) significantly reduced LPS-induced upregulation of Cox-2 protein which corresponds to the results reporting lack of CsA effect on LPS-induced Cox-2 expression in human microglial cells [25]. Inhibition of Cox-2 activity, either genetically or pharmacologically, had neuroprotective effects in rodent models of stroke, possibly via suppression of inflammatory reactions [26].

Inhibition of numerous microglial functions by immunosuppressants suggests blocking the early steps of microglia activation. However, the most described mode of FK506 action is inhibition of calcineurin, the inhibitory effects of other signaling pathways have been described, exemplified by inhibition of MAPK signaling in lymphocytes. In most cells, including glial cells, inhibition of calcineurin signaling is achieved with 1 μM CsA or FK506. As in LPS-stimulated microglia, the inhibitory effects are observed at ≥5 μM CsA or FK506; we suggest that inhibition of calcineurin is not a triggering event and rather inhibition of MAPK signaling, that is an early event and partly dependent on chaperone functions of immunophilins, plays more important role.

Here, we demonstrate that FK506 reduced MAPK phosphorylation in LPS-treated microglia that may be a potential mechanism of drug action. MAP kinases mediate microglial responses to LPS, cytokines, and growth factors, and control production of inflammatory mediators. MAPK inhibitors act as anti-inflammatory drugs by reducing synthesis of inflammation mediators at multiple levels and blocking inflammatory cytokine signaling [27, 28]. ERK, JNK, and p38 MAP kinases are activated by LPS in microglia and inhibition of a respective kinase with pharmacological inhibitors: CEP11004 or CEP-1347 reduced cytokine production in LPS-stimulated human and murine microglia cultures [29, 30] or exposed to neurotoxic peptide Aβ1-40 [30]. SP6001, an inhibitor of JNK, reduced LPS-induced *Cox-2*, *TNF-α*, *MIP-1*, and *IL-6* expression, while inhibitors of ERK1/2 and p38 attenuated LPS-induced cell enlargement [31]. Our results find confirmation in recent data showing inhibition of JNK activity in FK506-treated animals that links the neuroprotective action of FK506 with JNK signaling [32]. The inhibitory effect of FK506 on MAPK signaling may explain complex action, both on initiation as well as on propagation of inflammatory responses in microglia. Small molecule inhibitors’ targeting of p38 MAPK and JNK pathways showed a great potential as potent modulators of brain inflammation and gliosis in neurological disorders, where cytokine overproduction contributes to disease progression [27, 28, 33]. Inhibitors of p38 MAPK and ERK reduced IL-1β production, brain injury, and neurological deficits in cerebral focal ischemia [34, 35]. Studies with MAPK inhibitors on microglial cultures revealed distinct roles of particular kinase in controlling specific events in microglial activation [31], suggesting that a cocktail of MAPK inhibitors would be required to block efficiently all microglial responses. We demonstrate that FK506, a well-known drug with good pharmacokinetics, efficiently blocks many functions of microglia: cell enlargement, migration, and production of inflammation mediators at clinically relevant concentrations offering a new opportunity for treatment of poststroke neuroinflammation.

## Electronic supplementary material

Below is the link to the electronic supplementary material.

ESM 1 (XLS 72 kb)

ESM 2 (PDF 37 kb)

Supplementary Fig. 1. An effect of FK506/CsA treatment on naive microglia A. Morphological alterations of microglia exposed to 100 ng/mL LPS were not observed in cultures treated with immunosuppressants (at concentration 10 µM) for 24 h. Cells were stained with FITC-conjugated isolectin B_4_ (green) to visualize cell morphology followed by staining with DAPI for nuclei visualization (blue); 20X objective. B. Immunosuppressants do not induce migration of naive microglia in contrast to LPS. Confluent microglial cultures were scratched with a plastic 100 µL tip, washed with PBS and incubated for 5 hours under given conditions. Cells migrating to cell-free area without the treatment, after addition of 100 ng/mL LPS or immunosuppressants (10 µM) were visualized by phase-contrast microscopy (magnification x 4). C. Immunoblots show the levels of pro-IL-1β (31 kDa) and mature IL-1β (17 kDa), Cox2 and iNOS proteins in extracts from microglial cells 12 h after treatment with 100 ng/mL LPS or 20 µM immunosuppressants. β-Actin detection ensured an equal protein loading. Similar results were observed on 3 independently derived microglial cultures. (JPEG 349 kb)

High resolution image file (TIFF 4.79 mb)

Supplementary Fig. 2. Validation of microarray data on FK506 effect in the two systems compared: MCAo and LPS stimulated microglia by qPCR The expression of nine genes has been analyzed by quantitative PCR in independent in vivo experiments (the ipsilateral cortex of sham-operated, saline and FK506-treated ischemic rats at 12 h reperfusion, n=3) (A) and independent samples microglia (n=3) after 6 h of treatment with 5 µM FK506 and 100 ng/mL LPS (B). The amount of target mRNA was first normalised to the expression level of the *β-actin* mRNA amplified from the same sample and then to untreated controls for microglia or sham-operated samples for *in vivo* study, respectively. Gene expression changes are presented as fold change (relative quantitation) and are means +/- s.d. (n=3), ** p< 0.01, NS – not significant. (JPEG 235 kb)

High resolution image file (TIFF 525 kb)

Supplementary Fig. 3. 1 mg/kg FK506 administered 60 min after reperfusion reduces brain damage, microglia response and IL-1β production in the ischemic penumbra Representative microphotographs of Nissl staining (upper panel), lectin B_4_ labelled microglia (middle panel) and IL-1b immunostainig of the injured cortex of sham-operated, saline and FK506-treated at 24h of reperfusion. Scale bar: 250 µm in the upper panel; 100µ m in the middle and lower panels. (JPEG 431 kb)

High resolution image file (TIFF 6.42 mb)
