# Supplementary material for: Early steps of microglial activation are directly affected by neuroprotectant FK506 in both in vitro inflammation and in rat model of stroke
Source: J Mol Med (Berl). 2012 Jul 18;90(12):1459–71. doi: 10.1007/s00109-012-0925-9 (PMC3506835; doi:10.1007/s00109-012-0925-9)
Supplement: Supplementary file 2 — (PDF 37 kb) [file 109_2012_925_MOESM2_ESM.pdf]

Supplementary Methods

Quantitative Real Time PCR

Total RNA (2 µg) was isolated from microglial cultures or brain tissue exposed to various procedures using RNeasy kit (Qiagen) and used as a template. Amplifications were performed in duplicates in a 20-µl reaction volume containing 2xSYBR PCR MasterMix (Applied Biosystems) and a set of primers (sequences of PCR primers are listed in Supplemental Table 2). The amount of target mRNA was first normalised to the expression level of the *β-actin* mRNA amplified from the same sample and then to untreated controls for microglia or sham-operated samples for *in vivo* study, respectively. Data were analyzed by the Relative Quantification ($^{\Delta\Delta}$Ct) method using 7900 System SDS software (Applied Biosystems).

Histochemistry and Immunohistochemistry Procedures

The rats were deeply anesthetized with an overdose of sodium pentobarbital, and the brain was fixed by transcardial perfusion 4% paraformaldehyde (pH 7.4). Brains were immediately removed and postfixed for 1 day in the same fixative solution at 4°C. They were then placed in PBS containing 30% sucrose for 2 days at 4°C. The fixed brains were frozen in n-heptane at - 80°C and serial free-floating sections of 25 µm thickness were collected. Standard 0.1% cresyl violet staining (Nissl staining) was used to identify the site of the lesion and to evaluate the area of infarct.

For microglia detection, sections were incubated for 4 h at room temperature with peroxidase-conjugated isolectin B4 from *Bandeiraea simplicifolia* (10 µg/ml, Sigma, Germany). The peroxidase activity was visualized with 0.1 mol/L phosphate buffer containing 0.025% 3,3'-diaminobenzidine (DAB), 0.02% hydrogen peroxide and 0.6% ammonium nickel II sulfate hexahydrate. Nonspecific peroxidase activity was blocked by preincubating the sections in 3% hydrogen peroxide in 10% methanol at the appropriate step. Stained sections (bregma

0.20 mm, -0.40 mm, and -2.80 mm) were analyzed with an Olympus IX70 microscope and recorded photographically. The density of microglia was analyzed throughout five fields of cerebral cortex from each section. For IL-1β detection sections were preincubated in 0.1 M Tris-HCl and 0.9% NaCl (Tris-buffered saline, TBS) containing 2% BSA, 5% normal horse serum, and 0.3% Triton X-100 for 1 h, and then incubated for 48 h at 4°C with the addition of goat anti-IL-1β antibody (1:20, R&D Systems, USA). Bound immunoglobulins were detected by incubation with biotinylated horse antigoat IgG antibody (1:200, Vector Laboratories, Burlingame, CA) followed by avidin-biotinylated peroxidase complex (Vectastain Elite ABC kit, Vector). The expression of IL-1β was visualized by developing DAB reaction. The primary antibody omission controls were performed for each antibody.

Supplementary Table 2. Sequences of PCR primers.

Gene name	Forward primer	Reverse primer
β-actin	ACCACCATGTACCCAGGCATT	CCACACAGAGTACTTGCGCTCA
bdnf	GAGCGTGTGTGACAGTATTAG	GTAGTTCGGCATTGCGAGTTC
ccl-3	CATGGCGCTCTGGAACGAA	TGCCGTCCATAGGAGAAGCA
ccl-4	TATGAGACCAGCAGCCTTTGC	GCACAGATTTGCCTGCCTTT
ccl-20	GACTGCTGCCTCACGTACAC	CGACTTCAGGTGAAAGATGATAG
cxcl-2	ATCCAGAGCTTGACGGTGAC	AGGTACGATCCAGGCTTCCT
Il-1β	TACCTATGTCTTGCCCGGGAG	ATCATCCCACGAGTCACAGAGG
Il-6	AAATCTGCTCTGGTCTTCTGG	TTAGATACCCATCGACAGG
stat-3	GCCAATGCTGGAGGAGA	ACCAGCAACCTGACTTT
timp-1	GACCACCTTATACCAGCGTT	GTCACTCTCCAGTTTGCAAG

Supplementary Table 3. Contingency table, for the directions of the changes in expression in the two compared systems for the genes regulated in both systems.

		LPS vs MGCM		
		**Down**	**Up**	
**MCAo vs sham**	**Up**	FN: 187 (2)	TP: 392 (30)	579 (32)
	**Down**	TN: 223 (1)	FP: 41 (0)	264 (1)
		410 (3)	433 (30)	843 (33)

The left value in each cell is the count of all the regulated genes, the right value (in the parenthes) is the count of the regulated genes assigned to GO: inflammatory response.

The direction of the change in expression after the LPS stimulation was used as the test to predict the direction of the change following the MCAo. The positions of all the possible outcomes of these predictions are marked in the table: TP-true positives, TN-true negatives, FP-false positives, FN-false negatives.

Sensitivity=TP/(TP+FN)

Specificity=TN/(TN+FP)

<u>All the regulated genes (MCAo vs sham pval<0.05 and LPS vs MGCM pval<0.05)</u>

Fisher Exact Test

Uniwersum=33

Number of trials=579

Number of potential success=433

Observed number of successes=392

Two-sided p-value=1.3×10^{-47}.

Sensitivity=392/(392+187)=392/579=68%

Specificity=223/(223+41)=223/264=85%

<u>The regulated genes (defined as above) assigned to GO: inflammatory response</u>

Fisher Exact Test

Uniwersum=33

Number of trials=32

Number of potential success=30

Observed number of successes=30

Two-sided p-value=0.09.

Sensitivity=30/(30+2)=94%

Specificity=1/(1+0)=100%
