# Supplementary figures and images for: Early steps of microglial activation are directly affected by neuroprotectant FK506 in both in vitro inflammation and in rat model of stroke
Source: J Mol Med (Berl). 2012 Jul 18;90(12):1459–71. doi: 10.1007/s00109-012-0925-9 (PMC3506835; doi:10.1007/s00109-012-0925-9)

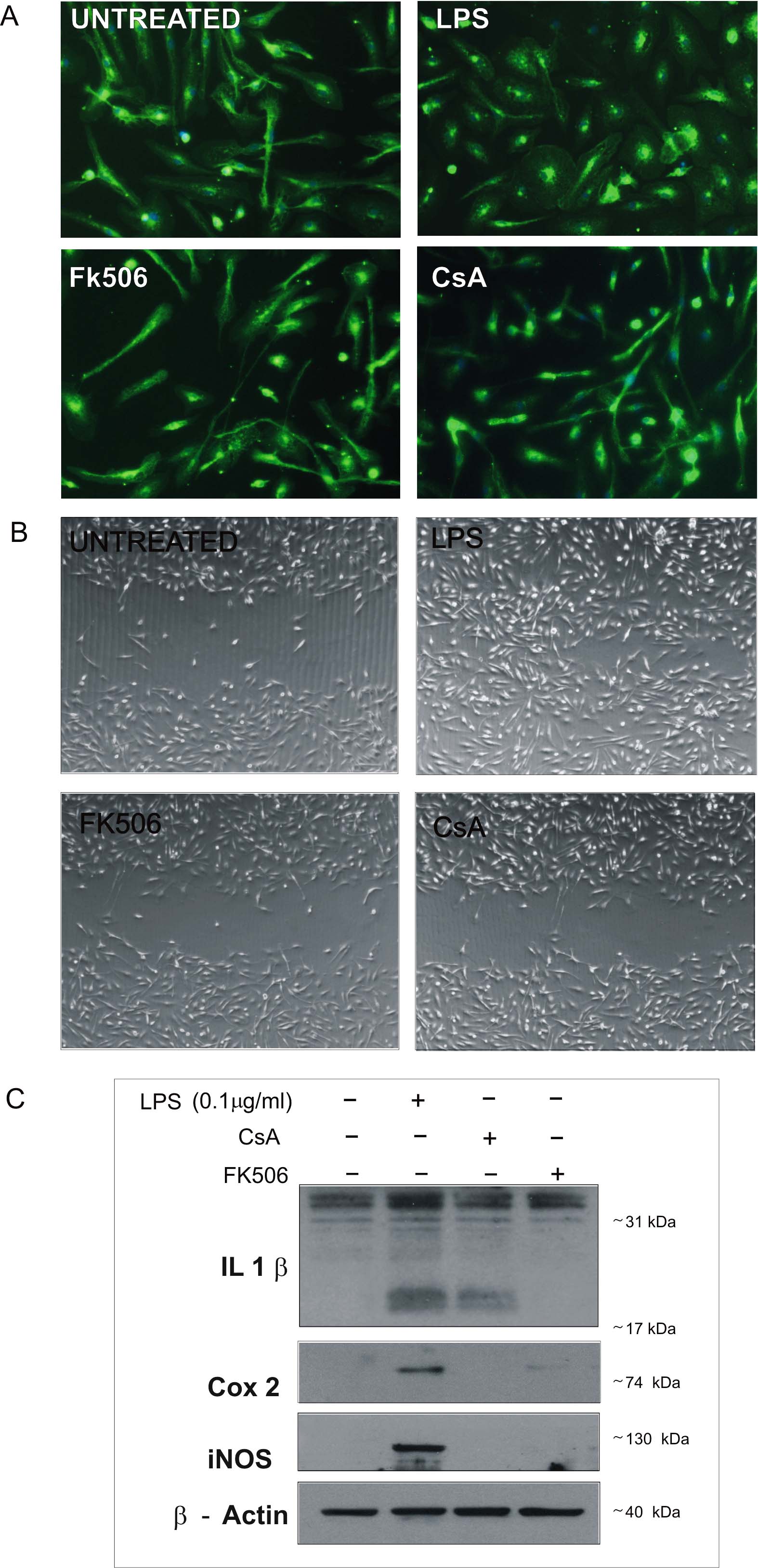

Supplement: Supplementary file 3 — A. Morphological alterations of microglia exposed to 100 ng/mL LPS were not observed in cultures treated with immunosuppressants (at concentration 10 µM) for 24 h. Cells were stained with FITC-conjugated isolectin B4 (green) to visualize cell morphology followed by staining with DAPI for nuclei visualization (blue); 20X objective. B. Immunosuppressants do not induce migration of naive microglia in contrast to LPS. Confluent microglial cultures were scratched with a plastic 100 µL tip, washed with PBS and incubated for 5 hours under given conditions. Cells migrating to cell-free area without the treatment, after addition of 100 ng/mL LPS or immunosuppressants (10 µM) were visualized by phase-contrast microscopy (magnification x 4). C. Immunoblots show the levels of pro-IL-1β (31 kDa) and mature IL-1β (17 kDa), Cox2 and iNOS proteins in extracts from microglial cells 12 h after treatment with 100 ng/mL LPS or 20 µM immunosuppressants. β-Actin detection ensured an equal protein loading. Similar results were observed on 3 independently derived microglial cultures. (JPEG 349 kb) [file 109_2012_925_Fig6_ESM.jpg]

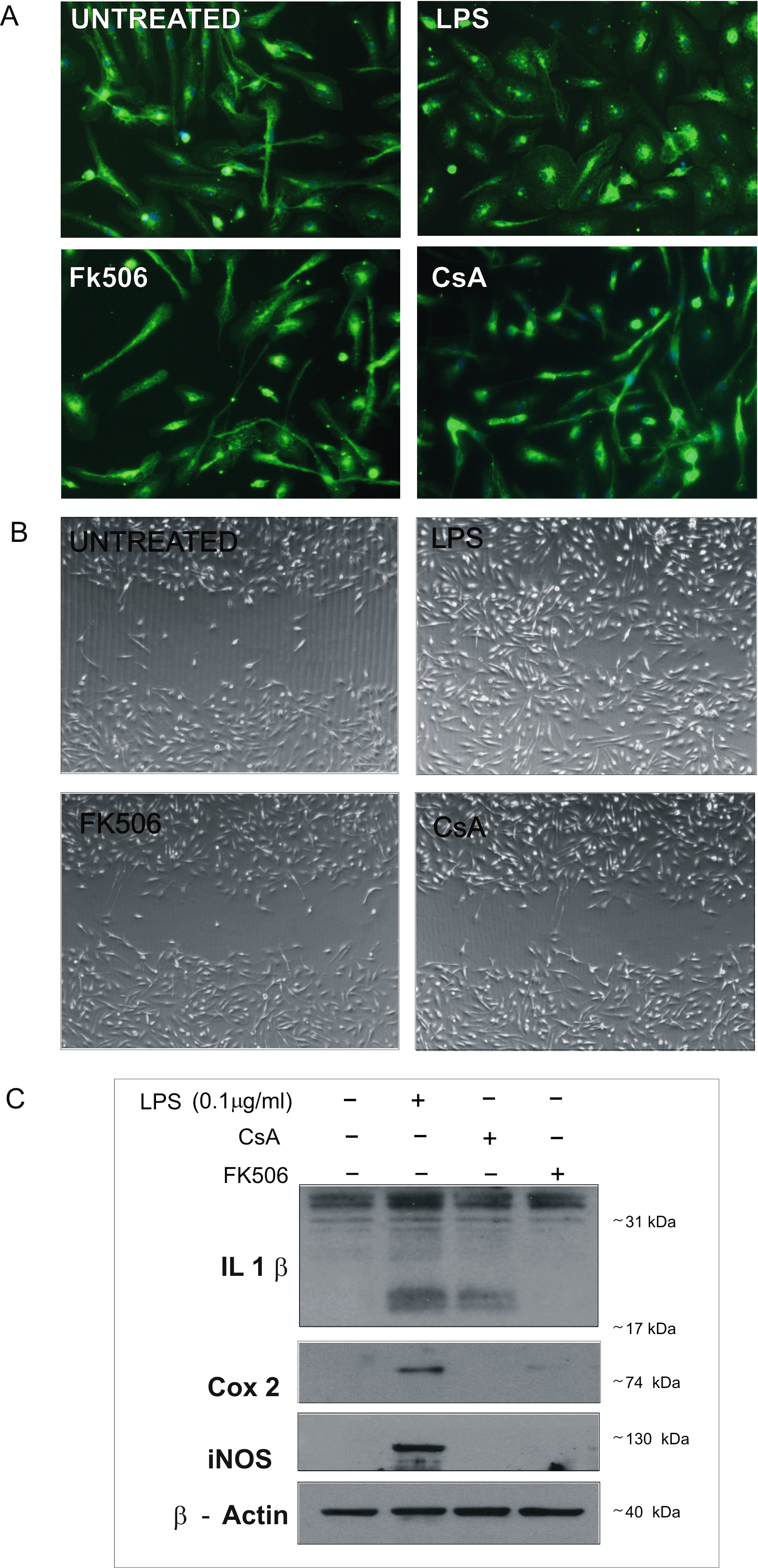

Supplement: Supplementary file 4 — High resolution image file (TIFF 4.79 mb) [file 109_2012_925_MOESM3_ESM.tif]

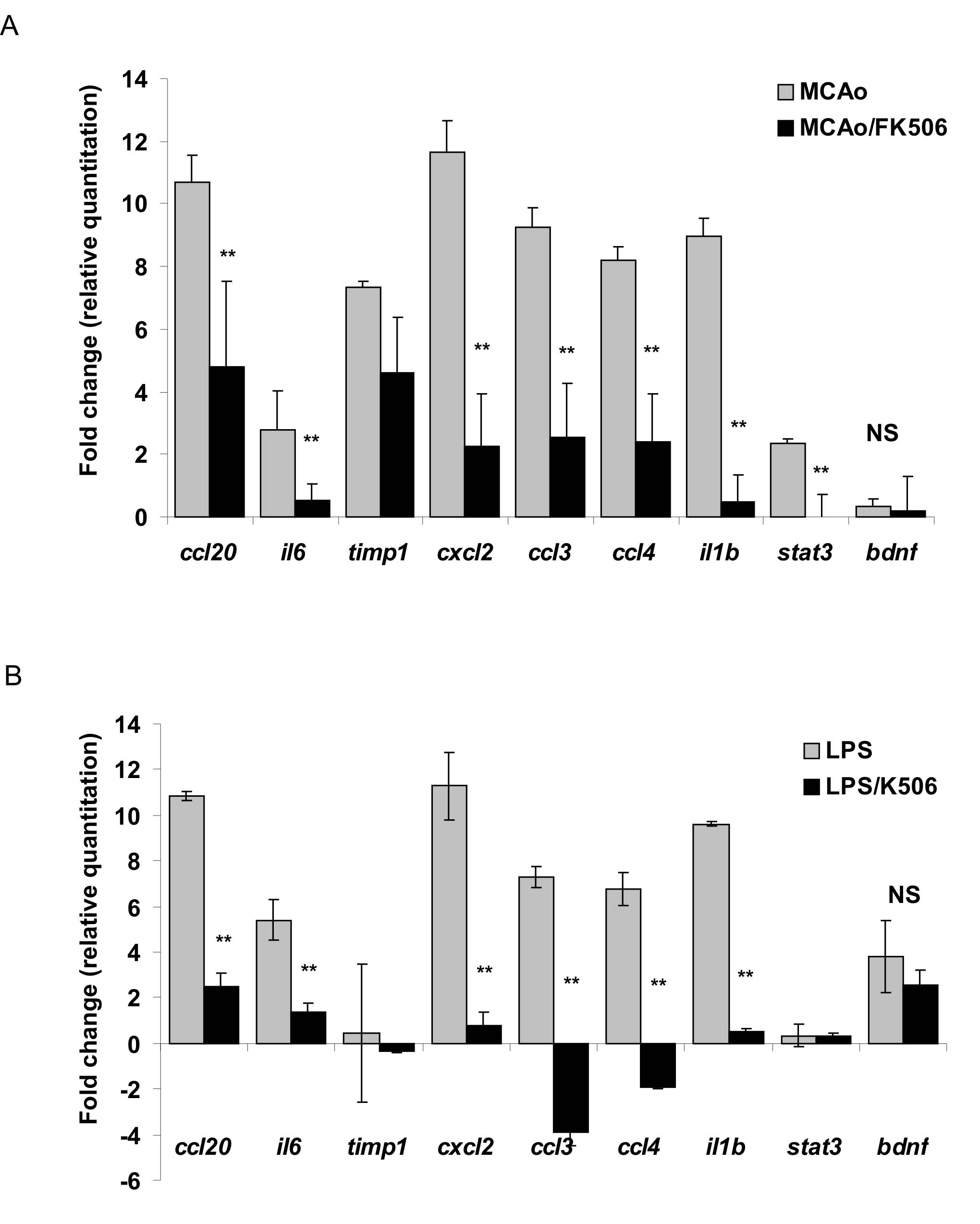

Supplement: Supplementary file 5 — The expression of nine genes has been analyzed by quantitative PCR in independent in vivo experiments (the ipsilateral cortex of sham-operated, saline and FK506-treated ischemic rats at 12 h reperfusion, n=3) (A) and independent samples microglia (n=3) after 6 h of treatment with 5 µM FK506 and 100 ng/mL LPS (B). The amount of target mRNA was first normalised to the expression level of the β-actin mRNA amplified from the same sample and then to untreated controls for microglia or sham-operated samples for in vivo study, respectively. Gene expression changes are presented as fold change (relative quantitation) and are means +/- s.d. (n=3), ** p< 0.01, NS – not significant. (JPEG 235 kb) [file 109_2012_925_Fig7_ESM.jpg]

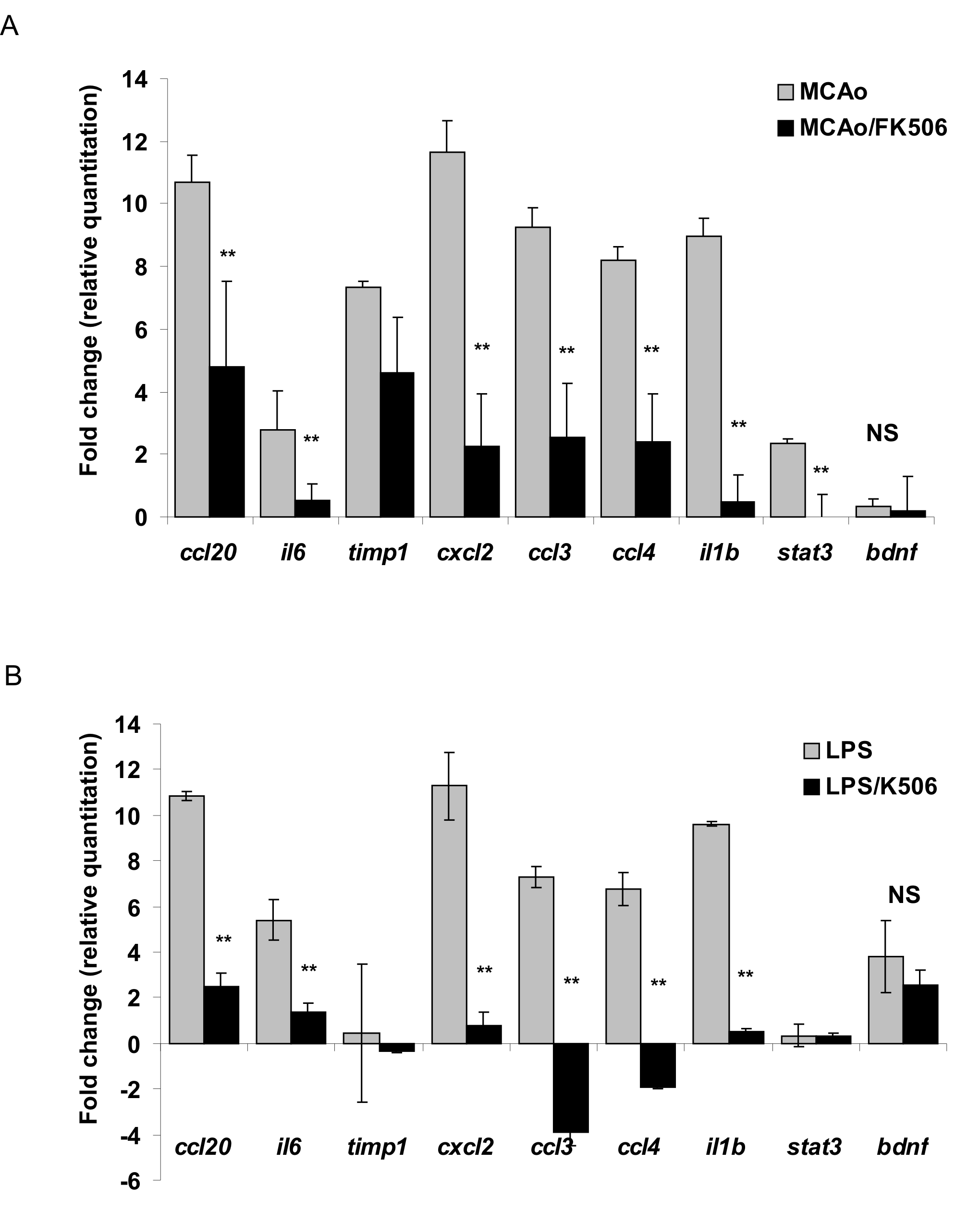

Supplement: Supplementary file 6 — High resolution image file (TIFF 525 kb) [file 109_2012_925_MOESM4_ESM.tif]

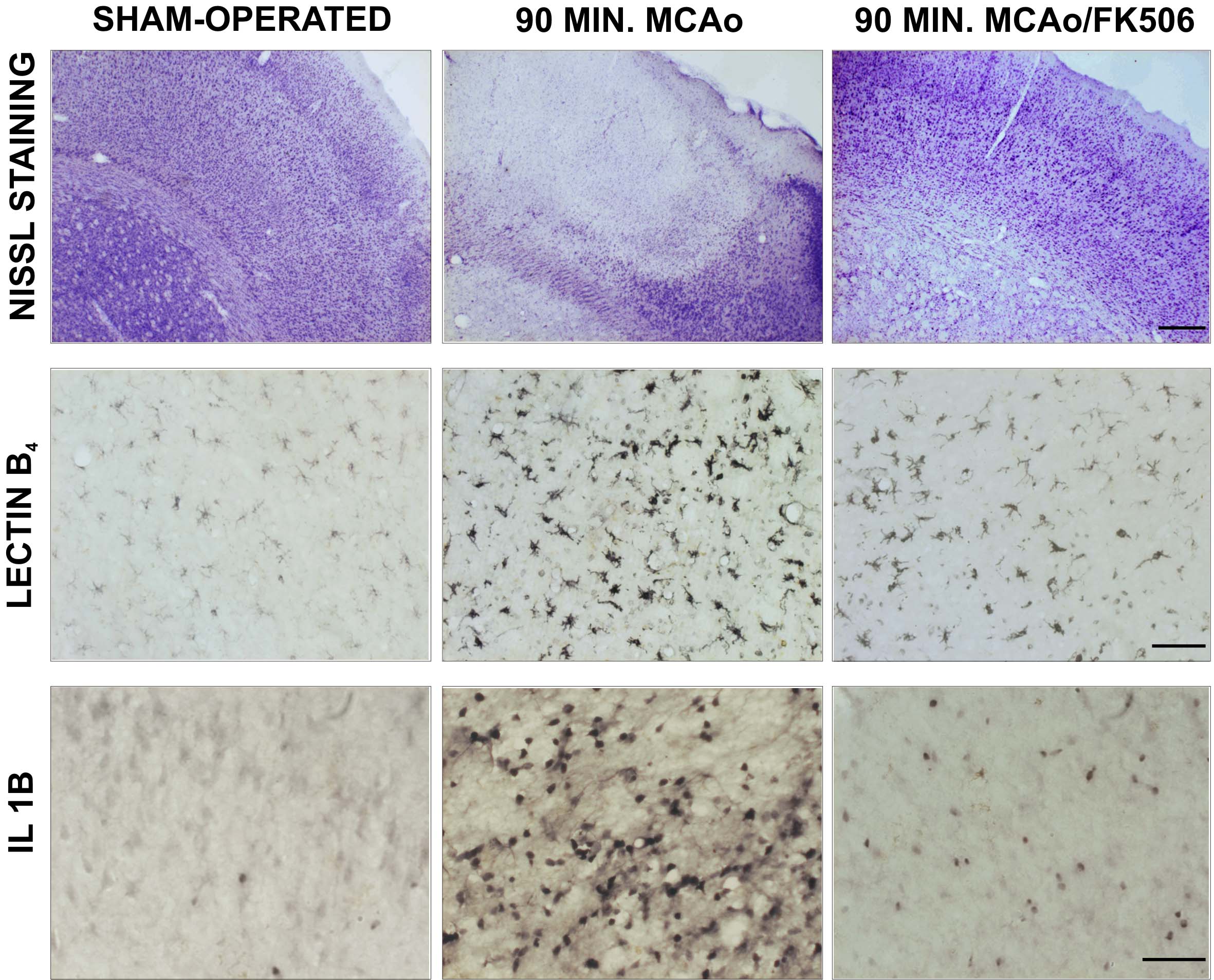

Supplement: Supplementary file 7 — Representative microphotographs of Nissl staining (upper panel), lectin B4 labelled microglia (middle panel) and IL-1b immunostainig of the injured cortex of sham-operated, saline and FK506-treated at 24h of reperfusion. Scale bar: 250 µm in the upper panel; 100µ m in the middle and lower panels. (JPEG 431 kb) [file 109_2012_925_Fig8_ESM.jpg]

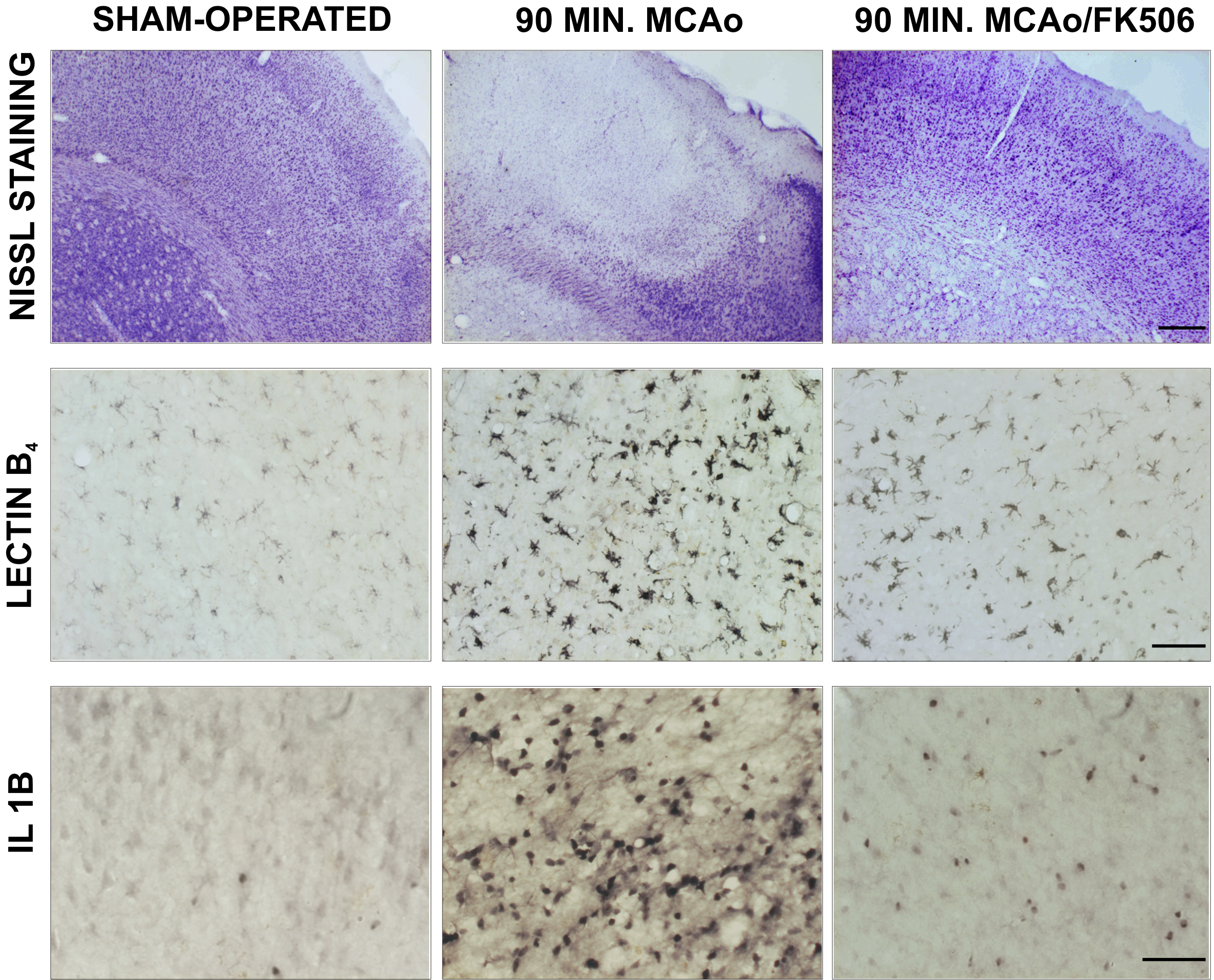

Supplement: Supplementary file 8 — High resolution image file (TIFF 6.42 mb) [file 109_2012_925_MOESM5_ESM.tif]
